# Effects of *Odontobuthus Doriae *Scorpion Venom on Mouse Sciatic Nerve

**Published:** 2013

**Authors:** Hossein Vatanpour, Amir Jalali, Edward G. Rowan, Fakher Rahim

**Affiliations:** a *Full Professor in Pharmacology and Toxicology, Dept. of Pharmacology and Toxicology, Pharmaceutical Research Sciences Center and School of Pharmacy, Shahid Beheshti University of Medical Sciences, Tehran, Iran.*; b *Associate Professor in Pharmacology and Toxicology Dept. of Pharmacology and Toxicology, School of Pharmacy, Toxicology Research Centre, Ahvaz Jundishapur University of Medical Sciences, Ahvaz, Iran.*; c *Full Professor in Pharmacology and Toxicology, Strathclyde Institutes of Pharmacy and Biomedical Sciences, University of Strathclyde, Glasgow, UK. *; d *PhD in Medical Genetics, Toxicology Research Centre, Ahvaz Jundishapur University of Medical Sciences, Ahvaz, Iran.*

**Keywords:** Mouse, Sciatic nerve, *Odontobuthus doriae*, Venom, Iranian scorpion

## Abstract

Temporary paralysis is a rare manifestation of envenoming following the yellow Iranian scorpion, *Odontobuthus doriae (O. doriae). *Thus, to elucidate the underlying mechanism, we investigated the neurotoxic effect of venom in the sciatic nerve, the possible mechanism in a mice model. The neurotoxicity and temperature effects in the venom-induced neurotoxicity were examined using the mouse sciatic nerve and mouse phrenic nerve-hemidiaphragm (MHD) preparations. *O .doriae *venom (1 μg/mL) caused changes in the perineural waveform associated with nerve terminal action potentials. Venom affected on both negative and positive components of the waveform which is known as a compound action potential. The timeresponse relationship of venom-induced depression of resting membrane potential (RMP) was significant (p < 0.05). No significant difference in augmentation was seen in room temperature in comparison with 37°C. In conclusion, although there was no evidence that the venom had any specific curarizing action at the neuromuscular junction, the results suggest that the venom exerts its neuromuscular transmission on the sciatic nerve through potassium and sodium ionic-currents. Furthermore, the influence of temperature on neurotoxicity was ineffective on blockade of the neuromuscular transmission *in-vitro*.

## Introduction

The venom of scorpions has potent neurotoxins affecting the neuromuscular transmission at either presynaptic or post-synaptic levels. Presynaptic-acting neurotoxins (β-neurotoxins)

inhibit the release of massive amounts of neurotransmitters, mainly acetylcholine and/ or noradrenalin, while post-synaptic-acting neurotoxins (α-neurotoxins) cause a reversible blockage of acetylcholine receptors (1, 2). These effects induce and cause central and peripheral neurotoxicity, cardiotoxicity and metabolic alterations (3-5). These toxins have an effect on the normal function of excitable tissues found in muscles and nerves by interaction with Na^+^ , K^+^ , Ca^2^^+^ or Cl^-^ channels (6, 7). These basic and low molecular-weight peptides are of great interest in neurobiology and pharmacology.

**Figure 1:**
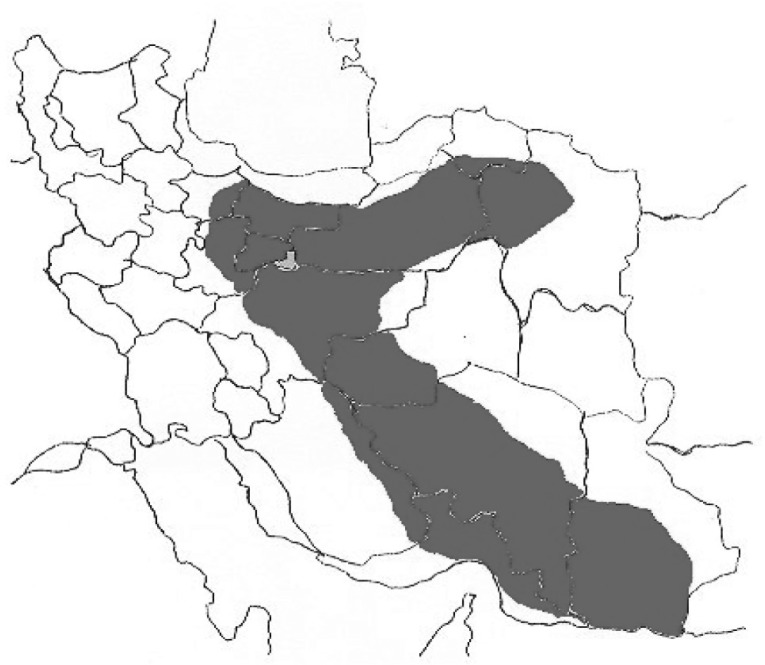
Geographic distribution of the yellow Iranian scorpion (O.doriae).

The Iranian yellow scorpion O. doriae is a member of the Buthidae family and is especially found in the central and southern part of Iran (Figure 1). The genus Buthus has two species: doriae and odonturus (8). O. doriae venom is able to interact with some voltage-dependent sodium (Na_v_) and potassium channels (K_v_) (9- 12). Thus far, a venom component has shown to be highly subtype-selective for a Na_v_ isoform, Nav1.7 (13).

Its stings cause various effects ranging from local pain, inflammation, necrosis, hematological effects (12), and neurotoxicity. The early neurotoxicity, within a few hours, develops rapidly, leading to a range of temporary paralysis. Temporary paralysis covers a range of injuries ranging from mild, moderate to severe, permanent paralysis, affecting as little as one muscle group to as much as the full body. Although this sign occurs rarely, almost in severe envenoming,underlying exact mechanism was needed to take into account. Considering the strength and wide spread of stings by this scorpion in center parts of Iran (Figure 1), a sciatic etiology remains the most probable, perhaps accentuated by individual susceptibility or age. Therefore, the present hypothesis was designed to raise the knowledge on the motor nerve terminal activity of the sciatic nerve following the inoculation of scorpion venom. In addition, in order to advance our understanding of the mechanism of neuromuscular transmission blockage *in-vitro*, the influence of temperature in the event of progression of neurotoxicity and complications of envenoming was investigated in the MHD preparation.

## Experimental

*Study design and population*

A total of 15 healthy, adult, clean grade BALB/c, male, mice, aged 8 weeks, weighing 25 ± 2 g, were provided. All mice were housed in groups of five and maintained under a 12-h light-dark cycle in a temperature-controlled environment (20 ± 22°C) for at least 10 days prior to the experiments. Food and water were freely available. Animal handling procedures were in accordance with the guidelines for animal care prepared by Committee on Care and Use of Laboratory Animal resources, National Research Council, USA.

*Materials*

Crude venom was obtained by electrical stimulating the telson of scorpion and after freeze-dried was stored at - 50°C until used.

*Mouse phrenic nerve-hemidiaphragm (MHD) preparation*

Mice (20-25 g) were killed using CO_2_ and decapitated. Hemidiaphragm and the attached phrenic nerves were dissected from the animals in the same way as described for the rat phrenichemidiaphragm preparation as described previously (14). The nerves were mounted in 5 mL tissue bath containing Krebs physiological solution at 37°C and gassed with 95% O_2_ + 5% CO_2_ (pH = 7.3). The composition of the Krebs physiological solution was as follows: NaCl, 118.4; KH_2_PO_4_, 1.2; glucose, 11.1; NaHCO_3_, 25; CaCl_2_, 2.5; MgSO_4_, 1.4 and KCl, 4.7. The preparations were mounted on an electrode enabled to stimulate the nerve directly. For indirect stimulation, the phrenic nerve was stimulated by pulses with a voltage greater than that required to produce a maximal response. A single pulse generated by an electronic stimulator (Grass S88B, Quincy, MA) was delivered to induce a twitch at a frequency of 0.1 Hz with pulses of 0.2 ms duration.

*Resting membrane potential recording*

Intracellular recordings of the Resting membrane potential **(**RMP) potential were performed on unstimulated fibers equilibrated in standard physiological solution. This solution was contained 100 μM tubocurarine to overcome any possible effect of venom due to acetylcholine release from nerve terminal.

 RMP was measured by standard microelectrode techniques (Fatt and Katz, 1951). Muscle fibers of mouse sciatic nerve were depolarized by exposure to venom (1 μg/ mL). The glass microelectrode filled with 3M KCl (resistance: 10-20 MΏ) was inserted into a muscle fiber at an endplate region. Recording sites were rejected if the membrane potentials were less than 60 mV on the initial implement or the membrane potentials varied by more than 10% during the first 20 min of control recording.

*Mouse sciatic nerve recording*

A 5-7 cm long sciatic nerve, comprising proximal and distal regions, was dissected out. The preparation was then mounted into a recording chamber consisting of three cylindrical compartments of 1 mL capacity each. Electrical isolation between the compartments was achieved using Vaseline. The two external compartments were filled with physiological buffer (15). The composition (mM) of physiological buffer was as follows: NaCl, 154; KCl, 5; MgCl_2_, 1.25; glucose, 11; HEPES, 5.46; CaCl_2_, 1.8; pH = 7.4. The central compartment contained the 1 mg/mL venom. Before each experiment the solution was saturated with 100% O_2_ for 30 min. Pellet type silver electrodes were dipped into each of the compartments of the recording chamber with stimulation taking place between the central and one of the external compartments. The ground electrode was dipped into the central pool. Recording were obtained from the central pool. A Grass S48 stimulator was used to supply electrical impulses (0.4 Hz, 0.04 ms duration) supra-maximally via a stimulus isolation unit, model SIU 5A (Grass Instrument Co., Quincy, Mass., USA). Signals were amplified with a CED 1902 transducer, digitized by an analogue-digital converter CED 1401 and analyzed with a microcomputer program (Dempster, 1988). Experiments were carried out at room temperature of 20 ± 2°C. The sciatic nerve preparation was incubated in physiological buffer for 30 min under constant super-maximal stimulation to demonstrate viability in the preparation and consistency in the recordings.

*Statistical analysis*

The measurements were expressed as the mean ± SEM (n = 3). Differences between groups or treatments were compared using Student’s t-test with p < 0.05 indicating significance.

## Results

*Effects of the venom on responses to indirect stimulation of mouse hemidiaphragm (MHD) preparations at room temperature*

**Figure 2:**
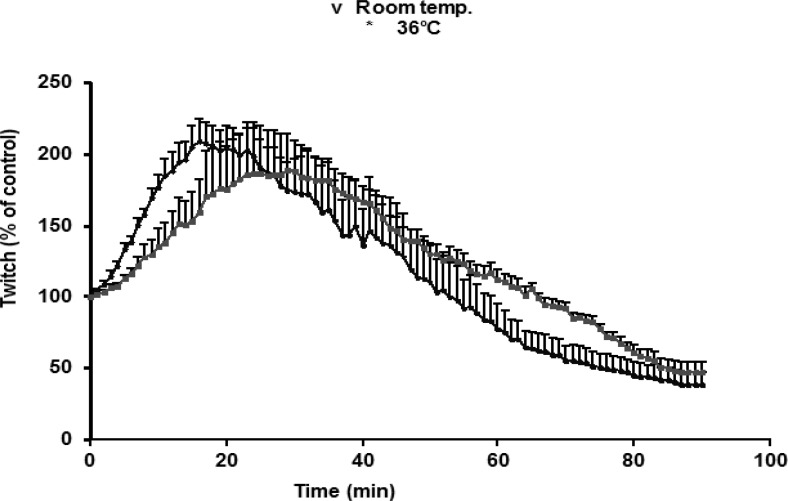
The effects of O.doriae venom (1 μg/ml) in responses to indirectly stimulation of MHD preparations at room (20±22˚C) and 36oC temperature. The ordinate represents the (%) amplitude of twitches relative to the initial amplitude. Each point is the mean ±S.E.M. of three muscles.

*O. doriae *venom, tested on indirectly stimulated preparations, caused a transient augmentation in twitches followed by a contracture, and then increasing in resting tension coincided with the reduction of twitch height until a complete irreversible suppression. A transient increase due to 1 μg/mL venom was slower at room temperature (20-22°C) compared with effects seen at 37°C. There was no significant difference in augmentation seen in room temperature compared with 37°C (Figure 2).

*Effects of the venom on resting membrane potentials of muscle fibers*

The RMP significantly decreased 80 and 60% after 10 and 30 min, respectively compared to control (p < 0.05; n = 3). RMP was decreased from - 75 ± 2.3 mV to - 60 ± 3.6 mV and – 45 ± 2.3, after 10 and 30 min, respectively. The depression was remained up to 40 min. The time-response relationship of venom-induced depression of RMP was significant (p < 0.05).

*Effects of venom on mouse sciatic nerve*

**Figure 3:**
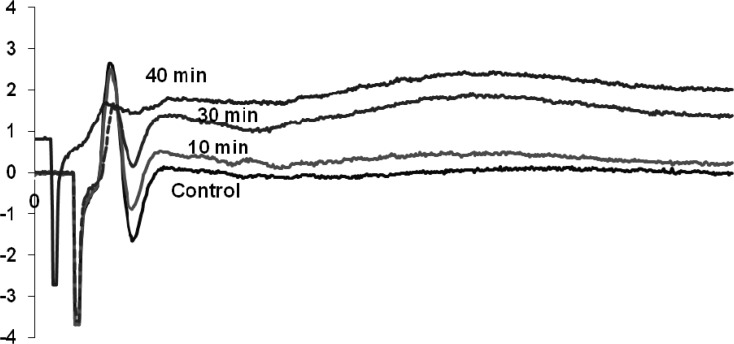
Compound action potential control and treated with 1μg/ml O.doriae venom recorded at 10, 30 and 40 min.

Venom (1 μg/mL) affected on both negative and positive components of the waveform which is known as a compound action potential. The venom after 40 min exposure abolished the negative part of the waveform and reduced the amplitude of the positive spike. Other characterization of the waveform remained similar to control (Figure 3).

## Discussion

To underlying the mechanism of action of temporary paralysis following an O. doriae sting, the experiments were performed on compound action potentials recorded from isolated preparations of mice sciatic nerve. Injury to the sciatic nerve causes weakness or paralysis of one hind leg in human and the animal is well known. So, we aimed to elucidate whether sciatic nerve neurotoxicity or reversible neuromuscular transmission blockage underlie this association.

The mouse hemidiaphragm (MHD) preparation as a neuromuscular junction is the physiologic target for many venoms or highly selective toxins acting presynaptically and/or post-synaptically on ion channels and receptors.

Therefore, it was of interest to determine the effects of venoms or toxins on isolated mouse MHD preparations. In MHD preparation, presynaptic effect of the venoms abolishes or reduces nerveevoked responses, without affecting responses to cholinoceptor agonist or KCl-induced contracture of the muscle. Postsynaptic receptor effects of venoms decrease or block responses of the muscle to nerve stimulation and cholinoceptor agonists as well, but would not affect responses to direct muscle stimulation or to elevated KCl. In contrast, myotoxin could reduce or block the responses of a skeletal muscle to direct electrical stimulation, to elevated extracellular KCl, to nerve stimulation and cholinoceptor agonist (16, 17).

*O. doriae venom *possesses highly specific toxicity towards neuromuscular preparations.

At the MHD preparation, venom acts primarily by causing triphasic changes (depression, facilitation and final blockade) of twitch tension of motor nerve terminal. There were also immediate contractures in muscle fibers, which probably were due to massive release of acetylcholine from the endplate. We described the effect of the venom on chick biventer cervicis preparations, in which inhibition of twitchtension and KCl-induced contractures was observed (11). High concentrations of venom and its myotoxic component were necessary to cause neuromuscular blockade. Indeed, as reported by Harvey *et al. *(16), low venom concentrations frequently expose the presence of neurotoxins, while high concentrations are required to demonstrate the presence of myotoxic components. Therefore, the concentration of 1 μg/mL of venom was selected for the experiments.

 The *O. doriae *scorpion venom reduced the amplitude of the second component of the sciatic preparation followed by abolishing the reverse phase. The perineural waveform in normal conditions consists mainly of two downward negative spikes, the first of which corresponds to the inward sodium currents at the final nodes of Ranvier, and the second of which reflects the outward flow of potassium ions at the distant nerve terminals (18). It completely abolished the second negative component of the waveform. Longer exposure caused a small decrease in the amplitude of the first positive component of compound action potential.

Studies have shown that the venom or toxins that reduce the second negative component able to facilitate the release of acetylcholine (19, 20). Suppression of the potassium current at nerve terminals would probably lead to a slower repolarization of the nerve terminal and consequently, allow calcium channels to open for longer durations. The effect of the venom in initiating muscle contractility has been attributed to interference with the stabilizing function of calcium at the muscle membrane (21).

*O. doriae scorpion venom *contains both α-like toxin OD1, which slow sodium channel inactivation, as well as toxins affecting on potassium channel, which causes a negative shift in the conductance-voltage relationship of the potassium channel (9, 10, 12, 13). So, *O. doriae *venom contains toxins, which act on ion channels causes either polarization or depolarization of excitable membranes such as sciatic nerve, which affects the action potential and neuromuscular function (22). In addition, venom prolongs the action potential of the sciatic nerve. These changes may increase the presynaptic release on neurotransmitters, including catecholamine (23), acetylcholine (24), glutamate (25), and GABA (26), contributing to the observed of neurotoxicity in *O. doriae *envenomed patients. This conclusion is augmented with no change in sciatic nerve preparation exposed to Conus geographus venom (27). The Conus geographus venom did not emerge to block sodium ion transport and did not affect the action potentials in the prepared nerve preparations. 

Since the effects of the venom were going to be tested in electrophysiological experiments at room temperature, the effects of venom (1 μg/mL) at 37°C were compared with those at room temperature. The rate of augmentation was slightly faster at room temperature than 37°C, although the pattern of augmentations was similar. Same effects have been studied experimentally with previous studies (20, 28, 29). These effects have been attributed to the dependence of transmission efficiency on temperature. As suggested by previous studies conducted on the snake venoms, the influence of temperature upon the toxins induced neuromuscular blockade strongly showed the involvement of the enzymatic activity of PLA_2_ in the snake venom neurotoxicity (30).

Our observation did not show a temperaturedependent blockade of the neuromuscular transmission in MHD preparation. So the results reported here constitute the first evidence that neurotoxicity caused by scorpion venom at the neuromuscular preparation level may not be dependent on the PLA_2_ enzymatic activity. It means that neurotoxicity of scorpion venom don›t related to the PLA_2_ as showed in snake venoms.

## Conclusion

It is not fully recognized whether *O. doriae *directly affects the sciatic nerve via channels or acts via neurotransmitters and inflammatory mediators. Based on the present results, O. doriae venom causes a strong depolarization of the channels, followed by a drop in excitability, leading to a temporary paralysis in the sciatic nerve. O. doriae toxins mainly Na_v_ isoforms prolong the action potential of the sciatic nerve and induce temporary paralysis. However, the influence of temperature on neurotoxicity was ineffective on blockade of the neuromuscular transmission *in-vitro*.
